# Understanding the nature of the Nef requirement for HIV-1 infectivity

**DOI:** 10.1186/1742-4690-10-S1-P75

**Published:** 2013-09-19

**Authors:** Annachiara Rosa, Massimo Pizzato

**Affiliations:** 1 Centre of Integrative Biology, University of Trento, Trento, Italy

## Background

Nef is an HIV-1 accessory protein with a fundamental role for virus replication *in vivo* and for the development of AIDS. Among its several activities, Nef is essential for maintaining HIV-1 maximal infectivity. In cell culture, such activity can account for as much as 98% of the HIV-1 virion infectivity and requires Nef to be expressed in virus producing cells rather than in target cells. However, the cause of the defective infectivity of Nef-negative HIV-1 and the mechanism by which Nef is able to restore such a defect remain elusive. The purpose of this research is to establish whether Nef contributes to HIV-1 infectivity by counteracting a cellular inhibitor or by promoting a cellular activity required for optimal infectivity.

## Materials and methods

Several human cell lines of different histological origin were used as HIV producer cells in order to study the variability of the requirement of Nef for infectivity. Single cycle infectivity was measured on a HeLa-based reporter cell line after normalization based on reverse transcriptase of the inocula. To determine the dominance of the Nef requirement for optimal infectivity, virus is produced from heterokaryons generated from cells responsive or not-responsive to Nef.

## Results

We analyzed the ability of Nef to enhance HIV infectivity produced in more than 50 cell lines. We found that the requirement of Nef is highly variable. We identified a group of cell lines (Nef-responsive) in which the lentiviral protein is most required (10-50 fold) and a group of cell lines in which Nef is not at all or only weakly required (Nef-unresponsive, max 2-3 fold). All Nef-responsive cell lines belong to the lymphoid lineage, in line with the lymphotropic nature of HIV, while most cell lines belonging to the Nef-unresponsive group include mostly non-lymphoid cells. However, we identified two Nef-unresponsive cell lines of lymphoid origin, providing useful tools for further investigation to study the dominance of the Nef requirement for infectivity. We have established a heterokaryon assay which allows HIV-1 production only upon fusion of producer cells expressing complementary parts of the virus. We are now in the process of coupling producer cell lines with opposite Nef-responsiveness in order to verify the possibility that Nef counteracts a dominant cellular inhibitor of retroviral infectivity predominantly expressed in lymphocytes.

## Conclusions

Our findings show that the Nef requirement for virus infectivity is extremely variable depending on the type of producer cells and is strongest in lymphoid cells. From the study of cell lines with marked differences for Nef-responsiveness, we aim to identify cellular factors which determine the Nef requirement for HIV infectivity.

